# Testing for allergic disease: Parameters considered and test value

**DOI:** 10.1186/1471-2296-9-47

**Published:** 2008-08-26

**Authors:** Sheryl L Szeinbach, Spencer E Harpe, P Brock Williams, Hanaa Elhefni

**Affiliations:** 1 Division of Pharmacy Practice & Administration, College of Pharmacy, The Ohio State University, Columbus, OH 43210, USA; 2 Department of Pharmacy, School of Pharmacy, Virginia Commonwealth University, Richmond, VA, USA; 3 Medical School, University of Missouri, Kansas City, MO, USA; 4 Macro International Inc., Bethesda, MD, USA

## Abstract

**Background:**

Test results for allergic disease are especially valuable to allergists and family physicians for clinical evaluation, decisions to treat, and to determine needs for referral.

**Methods:**

This study used a repeated measures design (conjoint analysis) to examine trade offs among clinical parameters that influence the decision of family physicians to use specific IgE blood testing as a diagnostic aid for patients suspected of having allergic rhinitis. Data were extracted from a random sample of 50 family physicians in the Southeastern United States. Physicians evaluated 11 patient profiles containing four clinical parameters: symptom severity (low, medium, high), symptom length (5, 10, 20 years), family history (both parents, mother, neither), and medication use (prescribed antihistamines, nasal spray, over-the-counter medications). Decision to recommend specific IgE testing was elicited as a "yes" or "no" response. Perceived value of specific IgE blood testing was evaluated according to usefulness as a diagnostic tool compared to skin testing, and not testing.

**Results:**

The highest odds ratios (OR) associated with decisions to test for allergic rhinitis were obtained for symptom severity (OR, 12.11; 95%CI, 7.1–20.7) and length of symptoms (OR, 1.46; 95%CI, 0.96–2.2) with family history having significant influence in the decision. A moderately positive association between testing issues and testing value was revealed (β = 0.624, *t *= 5.296, *p *≤ 0.001) with 39% of the variance explained by the regression model.

**Conclusion:**

The most important parameters considered when testing for allergic rhinitis relate to symptom severity, length of symptoms, and family history. Family physicians recognize that specific IgE blood testing is valuable to their practice.

## Background

With the prevalence of allergic rhinitis estimated at 21% – 23% for the European population and 20% – 40% for the western population, appropriate diagnosis and treatment of allergic rhinitis is of global importance [1,2]. Family physicians are usually first approached by patients experiencing symptoms; however, little information exists regarding the rationale to perform specific IgE blood testing, which parameters are most important, and the value of such testing. Given the need to determine if symptoms are truly attributed to allergic mechanisms, it is important that family physicians consider diagnostic testing in conjunction with a careful examination of patient history, clinical evidence, and environmental exposure factors to optimize patient care. The consequences of untreated symptoms can lead to multiple future complications while the consequences of misdiagnosis can lead to inappropriate treatments [3].

Chronic rhinitis has detrimental effects on quality of life and work productivity [4,5]. Although medications may control symptoms in some patients, it is difficult to distinguish between allergic rhinitis and non-allergic rhinitis using clinical evaluation and medication trials. Two commonly applied methods are used to uncover an allergic etiology and identify possible causes. These include skin prick tests (SPT), and specific IgE tests that are thought to produce concordant measures on a dichotomous basis for specificity and sensitivity, as well as a propensity toward appropriate diagnoses in relation to the presence of specific IgE antibody levels [6,7]. Decisions to utilize these tests are influenced by experience, patient history, diagnostic accuracy and efficacy of the test, and how well test results relate to symptoms [8,9].

When presented with patient complaints and bothersome symptoms that may or may not be related to allergic rhinitis, physicians rely on numerous strategies to make an appropriate diagnosis. How family physicians weigh the importance of these patient-related parameters when recommending specific IgE testing is largely unknown, yet instrumental to determine appropriate treatment and follow-up therapy. To address this research question, we used a trade off approach (conjoint analysis) to evaluate family physicians' preference to recommend specific IgE blood testing with respect to patient symptoms, family history, and medication use. A second approach using visual analog scaling (VAS) was added to validate and compare findings obtained from the conjoint analysis. Visual analog scales have been used extensively in clinical assessment to quantify patient perceptions of disease severity and the impact of symptoms on health [10,11]. Further evaluation was performed to determine if family physicians perceive that testing, as part of the care process was valuable to patient care. As healthcare gatekeepers, family physicians have the best opportunity to construct a baseline assessment of these patients to determine if current treatment strategies are effective, or if patients would benefit from a referral to an allergist or other specialist.

## Methods

### Study sample

Primary care (family) physicians in a southeastern state in the United States were identified through already established medical societies and physician mailing lists that were complied at the Recruitment and Retention Shared Facility at the University of Alabama, Birmingham. Mailing addresses, telephone numbers, fax numbers, specialty area, and practice affiliation was verified for 424 physicians in Alabama. From the list of 424 physicians, a sample of 150 physicians was randomly selected to participate in the study. Three separate mailings containing 50 questionnaires were sent by priority mail, one week apart to these physicians at their respective practice sites. The questionnaire package contained a letter of invitation to participate in the study, along with a self-addressed stamped envelope for the returned questionnaire. Thirty-two questionnaires were completed and returned during the first month, follow-up reminder calls were conducted three weeks after the initial mailing (77 calls were answered), and 14 surveys were faxed per request by providers. A total of fifty completed surveys were returned within two months. The estimated sample size of 50 was determined for this study following examples for studies with repeated-measures designs [12]. As an incentive, a gift certificate for a local department store was mailed to physicians who completed the questionnaire.

### Instrument Development

Techniques to evaluate preference include standard gamble, alternate rating (e.g., visual analog) scale, and time trade-off [13]. Besides these techniques, conjoint analysis (a trade off approach among attributes) is another technique that is used to evaluate the importance of preference measurements [14]. Choices are usually presented in the form of profiles that are ranked or rated (e.g., recommend specific IgE testing – yes or no). The part-worth values (coefficients) for each attribute are obtained from the random effects logistic regression model analysis with repeated measures (50 responses × 9 profiles = 450 observations), which follows stated choice experiments based on choice theory [15,16]. Although developed in marketing research, the use of conjoint analysis in health care is becoming a valuable tool [17-20].

In an attempt to simplify the conjoint exercise for this study, each attribute was assigned three levels (see Additional file 1). For example, symptom severity was assigned "high," "medium," or "low," and symptom length appeared as symptoms for "less than 5 years," "symptoms for 10 years," and "symptoms for more than 20 years." Family history included "neither parent has allergic rhinitis," "mother has allergic rhinitis," and "both parents have allergic rhinitis." Medication use included the use of "over-the-counter medications to control allergy symptoms," "prescribed antihistamines," and the "prescribed nasal spray to control allergy symptoms." Effects coding was used to construct the numerical values of the profile attributes. The "low" level was the reference value and was denoted as -1. The "high" level was denoted as +1.

A one-third fractional factorial design using repeated measures was chosen to minimize the number of profiles to 9 thereby attempting to avoid respondent fatigue. Two additional profiles were produced manually as holdout profiles for use in validation [21]. Each profile portrayed an individual with a pre-determined set of allergic symptoms and clinical indicators. For the dependent variable, family physicians were asked to provide a "yes" or "no" response to whether they would recommend specific IgE blood testing for this patient. For the purposes of this study, which specific IgE blood test was used by family physicians was not important, or what type of test (food or inhalant) was performed.

The hypothesis for this study was that the estimated part-worth values or coefficients, exponentiated to odds ratios in this study, for each of the four profile attributes were simultaneously equal to zero. In the next section, family physicians were asked to indicate if recommendations to test were influenced by managed care guidelines, value of testing, referral activities, familiarity with specific IgE testing, relation of test results to symptoms, and value of test to practice, the likelihood to use specific IgE testing using a ten-point scale "less likely to test" to "more likely to test." Specific IgE blood testing was rated using a scale ('1' = not valuable to '6' highly valuable) for overall value, value compared to skin testing, and value compared to not testing at all. Demographic characteristics of participating family physicians, such as age, gender, years in practice, and practice site information, were elicited in the last section of the questionnaire. To assess questionnaire validity, participants provided an estimate of a patient's overall health status given the impact of various symptoms and clinical indicators with 0 being the "Worst Possible State" and 100 being the "Best Possible State." The means for these items were compared to those rankings obtained from the conjoint exercise to offer additional information regarding testing.

### Data analysis

Descriptive statistics were provided for demographic variables. The conjoint exercise data were analyzed using a random effects logistic regression model. This type of model was chosen since it produces standard errors that account for the intra-individual correlation. Assumptions of normality, linearity, and equal variances among the items were evaluated to ensure appropriate interpretation of statistical analyses. All statistical analyses were performed using Stata/SE version 9 (College Station, Texas, USA). An Institutional Review Board from the University of Alabama, Birmingham, granted approval for the study.

## Results

### Demographics

Participating physicians (33% response rate) were more likely to be male, between 40 and 60 years of age and with about 20 years of clinical experience in a private practice setting (Table 1). Independent *t*-test revealed that among older physicians (> 50 years), those with 10 or more years in practice placed a greater value on specific IgE testing than not testing (n = 32; mean = 4.6) compared to those with less than 10 years in practice (*n *= 18; mean = 3.8; *t *= 2.2; *P *= 0.03).

**Table 1:** Demographic Characteristics of Family Physicians

Characteristic	Value (n = 50)
Age, y	
Mean (SD)	49 (12.0)
Range	29 – 79
	
Years in Practice	
Mean (SD)	18.6 (12.4)
Range	2 – 52
	
Gender, no. (%)	
Male	35 (71.4)
Female	14 (28.6)
	
Practice type, no. (%)	
Private/Independent	43 (87.8)
Managed care setting	6 (12.2)

### Conjoint model

Results from the random effects logistic regression model are presented in Table 2. The interaction between study attributes and demographic characteristics was not significant. Attributes, that are more likely to influence decision to request specific IgE blood testing, were symptom severity, length of time having symptoms, and history for allergic rhinitis reported for both parents. Results reveal the log likelihood = -196.983, Wald χ^2 ^= 94.03, *P *< 0.0001, with 448 observations for 50 physicians each physician evaluating 9 profiles, thus supporting the hypothesis that the impact of parameters on specific IgE blood testing are not perceived equally. Symptom severity had the greatest impact on physician decisions to test patients for allergic rhinitis (OR, 12.11; 95%CI, 7.1–20.7). Thus, one would expect that physicians would be 12 times more likely to consider the specific IgE blood test for patients with high symptom severity compared to patients with low symptom severity. Although not significant, other attributes such as length of symptoms and both parents having a history of allergic rhinitis influenced physician decisions to test (OR, 1.46; 95%CI, 0.96–2.2: OR, 1.44; CI, 0.95–2.2, respectively). However, some physicians may not be willing to trade among the alternatives when the decision involved a potentially dominant attribute, where symptom severity may be the only reason to recommend specific IgE blood testing. To assess the potentially dominant effect of symptom severity [22], two versions of the model were run – one containing profiles where symptom severity was present and one containing only those where symptom severity was absent (results not shown). In both situations, other parameter estimates were significant indicating the hypothesis that coefficients were simultaneously equal to zero was rejected regardless of the presence of the symptom severity.

**Table 2:** Results from the random effects logistic regression model

Attribute	Level	Odds Ratio	P-value	95% CI
Symptom severity	High	12.11	<0.001^a^	[7.1, 20.7]
	Medium	1.46	0.281	[0.84, 1.9]
	Low*	0.06		
Length of symptoms	>20 years	1.46	0.073^b^	[0.96, 2.2]
	5 years to 20 years	1.39	0.074^b^	[0.96, 2.2]
	<5 years*	0.47		
History of allergic rhinitis	Both parents	1.44	0.089^b^	[0.95, 2.2]
	Mother only	1.20	0.37	[0.80, 1.8]
	Neither parent*	0.58		
Medication use	Intranasal corticosteroids	1.12	0.586	[0.75, 1.7]
	Prescribed antihistamines	1.33	0.171	[0.89, 2.0]
	OTC allergy medications*	0.67		

*Baseline attribute level

^a ^p < 0.05; ^b ^p < 0.10

Log likelihood = -196.983; Wald Chi square = 94.03; p < 0.0001

448 observations for 50 individuals

### Validation

Two methods were used to validate the results of the conjoint exercise – the use of a holdout profile and an alternate rating method. First, was to estimate predictive validity for the holdout profile using the regression model developed from the 9 orthogonal profiles. The relation between the observed response for the holdout profiles and the predicted responses was then examined. The predicted values for the holdout profiles were quite similar to the observed value. The predicted mean probabilities were 82.7% and 78.4% compared to the observed values 70% and 78%, respectively. The differences were not significantly different (*t*-test; *p *= 0.162, *p *= 0.996, respectively) suggesting that the conjoint model exhibits acceptable internal predictive validity. Second, was the use of a VAS where participants responded to each item from the conjoint study presented separately. Lower mean scores obtained for each domain indicated that the particular domain represented choices that were less desirable to the respondent. Symptom severity (mean = 36.7; SD = 16.4) and symptom length (mean – 36.0; SD = 16.6) were ranked the worst followed by medication use (52.6; SD = 21.5), and family history (mean = 61.0; SD = 24.0), revealing consistent response patterns between the conjoint study and the VAS.

### Impact of testing issues on value

Kaiser-Meyer-Olkin measure of sampling adequacy for the final principal components analysis was 0.82 and the significant (*p *≤ 0.001) Bartlett test of sphericity supported the use of factor analysis for the items used to assess testing issues [23]. One factor was retained for testing issues accounting for 54.6% of the variance. Two items, difficulty in interpreting test results and insurance coverage were dropped from the analysis. The factor structure was further verified by reanalyzing the reliability of the dimension. Descriptive statistics (item's mean and standard deviation) and Cronbach's alpha for study items are presented in Table 3. Most noteworthy, was that physicians perceived that "how well the test correlated with symptoms" was given the highest score (mean = 7.6; SD = 1.9) with respect to "more likely to use specific IgE testing." In addition, physicians perceived that specific IgE testing had significant (*p *≤ 0.007) value overall, perceived value compared to not testing, and perceived value was comparable to skin testing. Cronbach's alpha for the remaining nine items for testing issues was 0.90 and 0.86 for the three items consisting of testing value, indicating a high degree of internal consistency or a high signal-to-noise ratio (i.e., error variance minimized) across individuals [24].

**Table 3:** Descriptive Statistics and Scale Evaluation for Issues and Test Value

	Mean (SD)
Testing issues *	
Managed care practice guidelines	5.4 (2.3)
Patient's perceived value of the test	6.5 (1.9)
Reduced need to refer patients to allergists	6.8 (2.0)
Difficulty in interpreting test results	4.4 (2.6)
Familiarity with test use	6.9 (2.3)
Patient demand to have the test done	7.0 (1.9)
Type of allergic rhinitis (intermittent vs. persistent)	6.6 (1.9)
How well test results relate to symptoms	7.6 (1.9)
Value of testing to my practice	6.9 (2.3)
Testing value **	
Overall value of specific IgE as a diagnostic tool	3.9 (1.1)
Compared to skin testing, usefulness of specific IgE blood testing	3.9 (1.4)
Compared to not testing at all, usefulness of specific IgE blood testing	4.3 (1.2)

* Issues – scale = 1 less likely to test, 10 = more likely to test; (measure of internal consistency of items – α = 0.90)

** Value – scale = 1 not valuable; 6 = highly valuable; (measure of internal consistency of items – α = 0.86)

Linear regression analysis was used to assess the relationship between testing issues and testing value. As hypothesized, results using composite scores for testing issues and testing value revealed a moderately positive association between these two dimensions (β = 0.624, *t *= 5.296, *p *≤ 0.001) with (R^2 ^= 0.39) 39% of the variance explained by the model.

## Discussion

According to our results, family physicians consider symptom severity to be the significant determinant, followed by symptom length and family history when recommending the use of specific IgE blood testing for patients suspected of having allergic rhinitis. Physicians in practice for 10 years or more placed greater value on specific IgE testing compared to those in practice for less than ten years. Moreover, results from VAS were consistent with findings from the conjoint study. Our findings were also corroborated in another recent study where VAS for symptom severity compared favorably with standard quality of life measures [25].

Professional organizations such as the American Academy of Allergy, Asthma, and Immunology and the European Academy of Allergology and Clinical Immunology recognize that allergic disease is a major health concern often requiring specific allergen avoidance and treatment strategies that are based on positive findings from history and diagnostic testing [26,27]. Results from this study support the positions elicited from the Joint Task Force on Practice Parameters for Rhinitis and Allergic Rhinitis and its Impact on Asthma (ARIA) in that family physicians are capable of recommending specific IgE testing, using the test to confirm allergic disease and identifying possible allergens [28-30]. Also consistent with recommendations from the Joint Task Force, results from the VAS closely approximated the findings of the conjoint study, thus revealing the usefulness of VAS in clinical practice to assess symptom severity for patients suspected of having allergic rhinitis.

Values for each item relating to patient perceptions of the test, patient demand to have testing performed, other clinical indicators, and the type of allergic rhinitis were summated to create a composite score. This composite score for testing issues yielded a moderately positive correlation with testing value, thus providing initial evidence that issues associated with testing and the process of care were linked to outcomes such as testing value. Moreover, positively framing the information describing the benefits of testing and the value of testing to patients is also known to influence their expectations of benefits [31].

Limitations include a low response rate and a cross-sectional study representing one geographical region. In addition, family physicians may consider attributes that were not evaluated in this study when deciding to request specific IgE blood testing for patients suspected of having allergic rhinitis. Hypothetical profiles were developed for this study and may not include all aspects of information provided by patients to family physicians, reflect what happens in actual clinical practice, and represent the opinions of physicians in other geographical areas.

Given the economic burden of allergic rhinitis on society and the research evidence that supports an inverse relationship between health status and specific IgE antibody levels [32-34], current guidelines should be repositioned and possibly modified to allow family physicians to have a more active role in specific IgE blood testing. Although ARIA suggests the SPT as a first line choice when further evaluation of patients is needed, interpretation of test results requires extensive training and experience. Thus, specific IgE testing was examined in this study as a practical choice for primary care physicians. As suggested from this study and supported in the literature, with proper training family physicians would become more adept at quantifying the results from specific IgE blood testing and recognizing when to refer patients (e.g., continued treatment failure, complications, and beyond scope of expertise) to allergists or other specialists [35-38]. Another important aspect of training is the need to consider specific IgE blood test and SPT results in the context of patient history, especially when discrepancy exists between test results and symptoms. Diagnostic testing, *per se*, is no substitute for a thorough examination of patient symptoms, health status, and medical history. In summary, allergists and family physicians understand that test results coupled with the findings of a careful clinical examination serve as the foundation to establish a strategy for treatment, from which future health outcomes can be evaluated to determine the success of treatment.

## Conclusion

Family physicians rely on symptom severity, and to some extent on length of time that symptoms are present and family history to determine whether patients should be tested to determine the presence of allergic disease. Physicians with more practice experience placed greater value on specific IgE testing. Findings also revealed a moderately positive association between the issues influencing the use of specific IgE blood testing and test value. Overall, family physicians valued specific IgE blood testing, especially compared to not testing.

From the study findings, family physicians can use symptom severity as a gauge in clinical practice to determine if patients should undergo detection and testing for allergic rhinitis or related conditions perhaps much earlier during the process of clinical evaluation, especially in the presence of severe symptoms and a positive family history. Baseline evaluation will also increase the likelihood of determining the correct diagnosis and appropriate treatment, and to ascertain the need for referral. Future research is needed to address the impact of patient expectations and treatment experience on value and other outcome measures.

## Competing interests

This study was supported by an unrestricted research grant from Phadia US Inc., Portage, Michigan.

## Authors' contributions

SLS conceptualized the study, examined the study design, performed the statistical analysis, and drafted the manuscript. SEH setup the study design, performed the statistical analysis, and drafted the manuscript. PBW conceptualized the study, examined the study design, and provided a critical assessment of the manuscript. HE coordinated and managed the collection of data for the study and reviewed the manuscript. All authors approved the manuscript.

## Pre-publication history

The pre-publication history for this paper can be accessed here:



## Supplementary Material

Additional file 1 Testing for allergic disease: Parameters considered and test value. Sample profile used for data collection in the conjoint exercise Click here for file
