# Supplementary material for: Testing for allergic disease: Parameters considered and test value
Source: BMC Fam Pract. 2008 Aug 26;9:47. doi: 10.1186/1471-2296-9-47 (PMC2532998; doi:10.1186/1471-2296-9-47)
Supplement: Additional file 1 — Testing for allergic disease: Parameters considered and test value. Sample profile used for data collection in the conjoint exercise [file 1471-2296-9-47-S1.doc]

**Scenario 3**

**1. Medium symptom severity**

**2. Symptoms less than 5 years**

**3. Both parents have allergic rhinitis**

**4. Uses prescribed antihistamines to control symptoms**

**I would recommend specific IgE blood testing for this patient: ____Yes ____No**
